# A Tailored Occupational Therapist–Led Vocational Intervention for People With Stroke: Protocol for a Pilot Randomized Controlled Trial

**DOI:** 10.2196/40548

**Published:** 2022-10-31

**Authors:** Sophie O'Keefe, Kathryn Radford, Amanda Farrin, Jodi Oakman, Serena Alves-Stein, Geoffrey Cloud, Jacinta Douglas, Mandy Stanley, Natasha A Lannin

**Affiliations:** 1 School of Allied Health, Human Services and Sport La Trobe University Melbourne Australia; 2 Department of Occupational Therapy Monash University Frankston Australia; 3 Nottingham University Nottingham United Kingdom; 4 Institute of Clinical Trials Research University of Leeds Leeds United Kingdom; 5 Centre for Ergonomics and Human Factors School of Psychology and Public Health La Trobe University Bundoora Australia; 6 Department of Occupational Therapy Alfred Health Prahran Australia; 7 Department of Neuroscience Monash University Melbourne Australia; 8 Department of Neurology Alfred Health Prahran Australia; 9 Living with Disability Research Centre School of Allied Health La Trobe University Bundoora Australia; 10 School of Medical and Health Science Edith Cowan University Joondalup Australia; 11 Alfred Health Prahran Australia

**Keywords:** return to work, vocational rehabilitation, acquired brain injury, stroke, traumatic brain injury, neuroscience, rehabilitation, intervention, feasibility, stroke recovery, resume work

## Abstract

**Background:**

Resuming work after stroke is a common goal of working-age adults, yet there are few vocational rehabilitation programs designed to address the unique challenges faced following stroke. The Work intervention was developed to address these gaps.

**Objective:**

This paper presents a protocol that outlines the steps that will be undertaken to pilot both the intervention and trial processes for the Work trial.

**Methods:**

The Work trial is a 2-arm, prospective, randomized, blinded-assessor study with intention-to-treat analysis. A total of 54 adults of working age who have experienced a stroke <4 months prior will be randomized 1:1 to either (1) an experimental group who will receive a 12-week early vocational intervention (Work intervention) plus usual clinical rehabilitation or (2) a control group who will receive only their usual clinical rehabilitation.

**Results:**

Outcomes include study and intervention feasibility and intervention benefit. In addition to evaluating the feasibility of delivering vocational intervention early after stroke, benefit will be assessed by measuring rates of vocational participation and quality-of-life improvements at the 3- and 6-month follow-ups. Process evaluation using data collected during the study, as well as postintervention individual interviews with participants and surveys with trial therapists, will complement quantitative data.

**Conclusions:**

The results of the trial will provide details on the feasibility of delivering the Work intervention embedded within the clinical rehabilitation context and inform future trial processes. Pilot data will enable a future definitive trial to determine the clinical effectiveness of vocational rehabilitation when delivered in the early subacute phase of stroke recovery.

**Trial Registration:**

Australian New Zealand Clinical Trials Registry ACTRN12619001164189; https://www.anzctr.org.au/Trial/Registration/TrialReview.aspx?id=378112&isReview=true

**International Registered Report Identifier (IRRID):**

DERR1-10.2196/40548

## Introduction

### Background

After a stroke, returning to work is challenging, and many people never return to employment [1]. Stroke leads to physical limitations (such as walking), cognitive impairments (such as memory or reasoning difficulties), as well as emotional and psychological challenges (including depression and apathy). Together, these limitations can result in challenges in returning to usual activities, especially work [2,3]. Nearly one-third of all strokes occur in adults who are of working age, and as many as 60% of people identify an unmet need with regard to work after stroke in Australia [4].

Resuming work is important for stroke survivors [5,6]. Evidence supports the benefits of working, including the economic resources to enable participation in society, meeting essential psychosocial needs, and being central to a person’s identity, social roles, and social status [6,7]. Conversely, there is a well-established association between unemployment and poor health in terms of higher mortality, poorer general health, and poorer mental health [8]. Unfortunately, at present, stroke survivors have limited and variable access to vocational rehabilitation that supports reintegration to work or rehabilitation for work-related activities [5,9]. Community rehabilitation, where available, rarely addresses needs beyond the traditional model of rehabilitation (gait, hand-therapy, speech, cognitive, and self-care rehabilitation) [5,9]. Such models of rehabilitation (focusing on the short-term delivery of rehabilitation interventions) may reduce impairments such as mobility but do not provide stroke survivors with the skills to confidently return to participating in important, everyday activities such as work. In other words, traditional rehabilitation does not currently equip stroke survivors to transition successfully from the rehabilitation environment to the work environment.

It is likely that early vocational rehabilitation, including work ability (how well someone’s health, skills, and experience match the demands of their work role) assessment, work visits, and the involvement of the employee, health professionals, and the employer, has the potential to improve participation in work for stroke survivors [2,10]. Yet it has become accepted that vocational rehabilitation is not a standard feature of poststroke rehabilitation programs [5,9]. Opportunities to provide vocational rehabilitation alongside clinical rehabilitation were an important consideration for the development of the Work intervention.

### The Work Intervention: An Occupational Therapist–Led Vocational Intervention for People With Stroke

The Work intervention is an individually provided, vocational intervention that is tailored to each participant’s return-to-work goals so as to enhance work ability. A total of 12 weekly sessions (1 hour per session) in either the inpatient or community setting commence with assessing the participant’s role as a worker alongside their stroke-related impairments. The occupational therapist then compares the client’s capabilities to the worker role requirements to forecast potential challenges that may be faced in the workplace; determine work limits and capabilities; coordinate appropriate supports and resources required from health care professionals, employers, and community services; as well as negotiate workplace adjustments, monitor return to work, and explore alternatives where current work is not feasible or sustainable [10-12]. Rehabilitation interventions then commence with establishing a daily routine in the home and building awareness of the worker role before all participants receive rehabilitation to address core work skills (such as problem solving complex situations or working as part of a team) [13]. The intervention may also address poststroke fatigue and enable community access through transport training or provide work conditioning to restore physical and cognitive capacity for work, work hardening programs [14], functional or cognitive capacity evaluations [15], or job task training before establishing an individualized return-to-work plan for each participant. The employer and relevant occupational health and safety staff from the workplace may be involved in the vocational rehabilitation program with permission from the participant, or the participant may only want to accept advice only about employer liaison.

To date, there has been no evaluation of the Work intervention, which was developed to be provided early after stroke (ie, within the first 4 months following a stroke while the participant is also receiving traditional clinical rehabilitation). Therefore, the aim of this pilot randomized controlled trial is to test the feasibility and potential benefit of adding a 12-week, stroke-specific vocational rehabilitation intervention to standard (clinical) rehabilitation, in either an inpatient or community context. The specific research questions posed in this pilot study are as follows:

1. What is the feasibility of adding early vocational rehabilitation alongside usual care for patients after a stroke?
2. What are the benefits of adding early vocational rehabilitation after stroke to those returning to work? Does it increase the number of working hours and improve confidence to work and self-reported quality of life?

While the main purpose of this study is to determine feasibility, regarding the effect of the Work intervention, we hypothesize that participants who receive vocational rehabilitation will experience higher rates of vocational participation and improved quality of life compared to the control group.

## Methods

### Study Design and Approach

**Figure 1:**
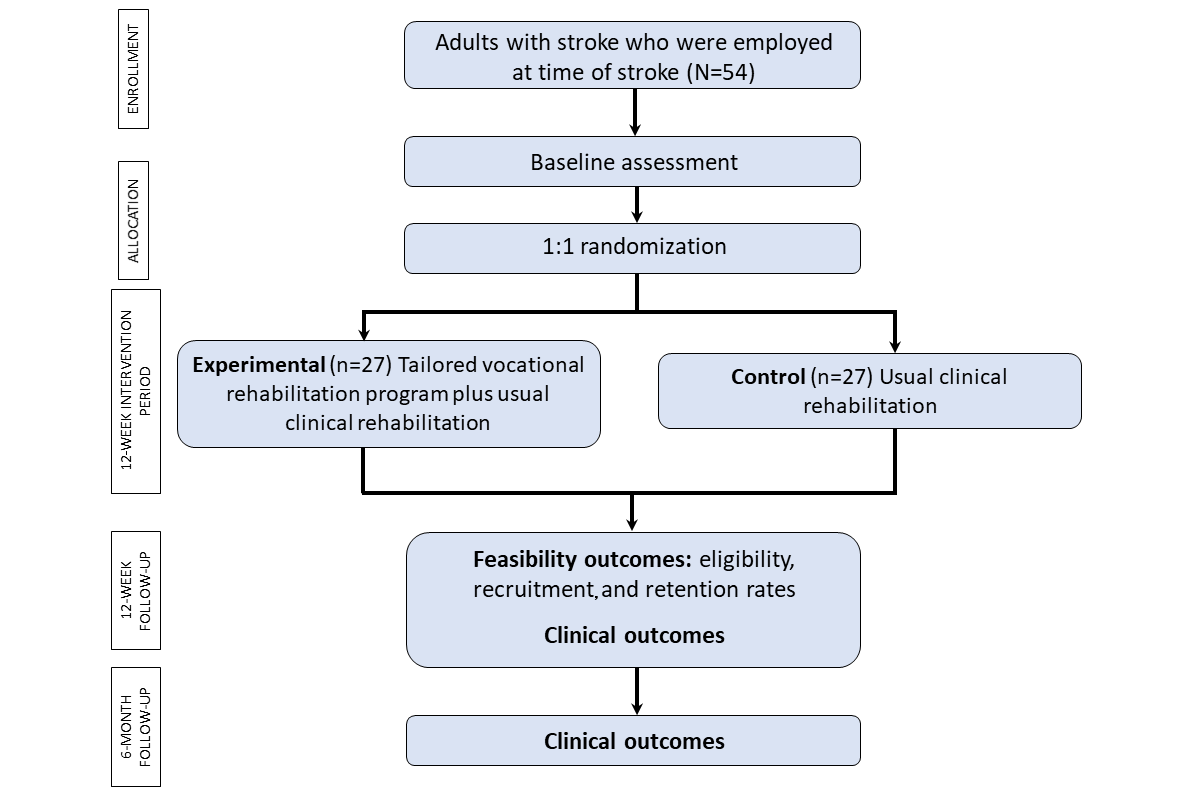
The CONSORT (Consolidated Standards of Reporting Trials) diagram of the Work trial.

The Work trial is an observer-blinded, pilot randomized controlled trial with concealed allocation, blinded measurement, and intention-to-treat analysis. We will recruit stroke survivors from acute, rehabilitation, and community settings at 1 health service in Australia. Participants will receive either (1) early vocational rehabilitation plus usual clinical rehabilitation or (2) usual clinical rehabilitation alone. Outcomes will be assessed at baseline, 3 months (end of intervention), and 6 months from randomization. Assessments will be collected by researchers blind to group allocation; it is not possible to blind participants or therapists to group allocation. Researchers blinded to group allocation will analyze the data. The design of the trial is presented in Figure 1.

### Ethics Approval

The Alfred Health Human Research Ethics Committee approved this study (HREC490/19, SERP56225; date of authorization: October 30, 2019), and La Trobe and Monash Universities’ human research ethics committees provided reciprocal registration. All participants will provide informed consent before data collection. This protocol was prospectively registered with the Australian and New Zealand Clinical Trials Registry (ACTRN12619001164189; date of registration: August 20, 2019).

### Consumer and Community Engagement

The views of people with lived experience of a stroke have been sought from the inception of this trial, including during a review of the funding application and protocol and selection of the primary outcome, and as representatives on the expert panel who will have oversight across the lifetime of the research. The expert panel consists of stroke survivors with lived experience of undertaking vocational rehabilitation as well as the failure to sustain working post stroke. The expert panel will continue to meet at least 3 times per year to provide input into the trial processes, documents, and intervention resources. These experts will be invited to review preliminary findings at the completion of data collection and will also inform dissemination strategies based on the pilot trial’s findings. All expert panel members receive a gift voucher following each consultation as recompense for their attendance at meetings.

### Participants and Therapists

Participants will be included in the study if they have been admitted to Alfred Health, a large tertiary hospital in Melbourne, Australia, with a new stroke in the last 4 months and have an identified vocational goal. Vocational goals will be inclusive of paid work, voluntary positions, and study, and be defined as being on sick leave due to stroke, unemployed, or underemployed. Participants will have the potential for a return to competitive employment within 6 months, which will be clinically determined based on a history of competitive employment within the last 3 years and a stated goal of returning to work.

People will be excluded if they do not speak English, have >10 years of formal education, have a history of nonstroke neuropsychological disorder resulting in cognitive impairment (eg, vascular dementia, brain injury, or Alzheimer disease), or have a receptive language or cognitive impairment significant enough to prevent the person from participating in the intervention. To determine the severity of cognitive impairment, the Oxford Cognitive Screen will be administered.

Therapists will be eligible to deliver the experimental intervention if they are registered occupational therapists with at least 3 years of experience in stroke rehabilitation. All therapists will be trained in vocational assessment and the intervention before intervention delivery by authors SOK and NAL.

### Randomization

Following baseline measurement, participants will be randomized via a web-based randomization program into 1 of 2 groups (experimental or control) using a process of minimization. Minimization will aim to balance 3 factors: baseline return-to-work status (unemployed or underemployed), age, and gender. The allocation sequence has been generated and will be managed by the Leeds Randomization Service at Leeds University and overseen by author AF.

### Intervention

#### Usual Clinical Rehabilitation

Both the control and experimental groups will receive usual clinical rehabilitation, which will be determined by their treating clinicians. Usual clinical rehabilitation may involve inpatient or outpatient/community physiotherapy, speech pathology, occupational therapy, psychology, or medical follow-up. Within the services involved in the pilot, the clinical teams work with the participant (and those important to them) to develop a rehabilitation plan directed by goals set by the participant and deliver interventions [16] that maximize their potential and independence [17].

#### Experimental Group Intervention: Early Vocational Rehabilitation Plus Usual Clinical Rehabilitation

Participants randomized to the experimental group will receive early vocational rehabilitation (the Work intervention) to address individualized goals set in relation to work and working in addition to usual clinical rehabilitation. The vocational intervention will be predominantly delivered face to face, but there is potential to deliver using telehealth [18]. Whether in-person or via telehealth, the intervention will be delivered on a one-to-one basis and be individually tailored to the participant in terms of content, dose, intensity, and duration according to participants’ needs and preferences (eg, whether the participant consents to employer liaison and workplace visits).

### Outcome Measures

Outcome measures will be collected by a health professional who is trained in the procedures and blinded to group allocation. Participants will be asked not to discuss any aspect of the trial with the assessor to protect assessor blinding.

#### Feasibility

Feasibility of the study will involve examining recruitment; adherence, acceptability, and safety of the intervention; and measurement of outcomes.

Feasibility of recruitment will be determined by calculating the number of enrolled participants as a proportion of the eligible population of stroke survivors who were working prior to admission to a hospital with a new case of stroke and retention of participants. Feasibility of providing early vocational rehabilitation will be determined by examining participant adherence to the intervention. Acceptability will be determined at the end of trial participation when each participant will be asked the following question: which intervention(s) would you prefer given the choice? Safety will be determined by recording injurious events. Feasibility of measurement will be determined by being able to measure the clinical outcomes in all participants.

#### Clinical Outcomes

All clinical outcomes will be assessed at each time point, that is, baseline, end of the intervention period (3 months), and follow-up (6 months), by a measurer who is blind to group allocation. The primary outcome is participation level in work, defined by the proportion of hours worked against a standard working week of 38 hours per week (7.6 hours per day) as well as days worked in the month prior.

Secondary outcomes include the following:

- Quality of life will be measured using the EuroQol Group’s EQ-5D-5L [19]
- The presence of anxiety and depressive symptoms will be measured using the Hospital Anxiety and Depression Scale [20]
- Social functioning will be assessed using the Work and Social Adjustment Scale [21]
- Functional independence to perform self-care and community activities will be assessed using the Autonomy Measurement System [22] and the Nottingham Extended Activities of Daily Living Index [23]
- Global disability arising from stroke will be rated using the modified Rankin Scale [24]

### COVID-19 Safety Measures and Contingency Planning

The Australian government and institutional COVID-19 safety procedures and public health regulations will be adhered to at all times. This includes: (1) pausing recruitment during periods of lockdown or stay-at-home orders; (2) having current COVID-19 safety plans in place for all research and community sites; (3) participant screening for COVID-19 symptoms, maintaining physical distancing requirements, cleaning and hygiene practices in line with infection control requirements, and use of personal protective equipment at all times. All staff will undertake training in COVID-19 safety procedures (Alfred Health, Melbourne, Victoria). With respect to extenuating circumstances leading to unplanned methodological, ethical, and/or analytical changes, protocol modifications will be submitted for approval. We plan to report modifications using CONSERVE (CONSORT and SPIRIT Extension for RCTs Revised in Extenuating Circumstances) alongside the CONSORT (Consolidated Standards of Reporting Trials) statement.

### Data Safety

Data safety and monitoring will be overseen by a health professional independent of the trial who will be responsible for reviewing all adverse events and ceasing of recruitment in the case of multiple, trial-related serious adverse events. For the purpose of this study, a serious adverse event will be defined as an event that (1) is life-threatening or results in death, (2) requires or prolongs existing hospitalization, or (3) results in persistent or significant disability. Adverse events will be monitored throughout the study period and compared across the intervention and control groups.

### Sample Size Estimates

Formal power calculations for feasibility studies are not usually undertaken; however, as the study is randomized, and we wish to obtain a preliminary estimate of effectiveness in relation to demonstrating how the intervention affects return-to-work rates, we propose a sample size that will be adequate to estimate parameters such as recruitment rate and sample variability for a phase III trial [25]. Based on these recommendations, we therefore propose to pragmatically recruit up to 54 participants.

### Statistical Analyses

Descriptive statistics will focus on CI estimation rather than formal hypothesis testing. Eligibility, consent, and recruitment rates will be reported to determine the acceptability of randomization. Reasons for ineligibility, nonconsent, and nonrandomization will be summarized. Rates of retention in, adherence to, and completion of treatment will be summarized by group. Follow-up rates and compliance with outcome measurement will similarly demonstrate the acceptability of the outcome measures. Reasons for dropout will also be summarized where possible. Interventions provided to all participants (ie, both groups) will be descriptively summarized. We will also report the difference and its CI for follow-up rates between the intervention and control groups to identify large differences between the two arms. Dropout rates over time will also be reported. Levels of missing data for outcomes will be summarized and compared between groups. All outcome measures will be summarized using appropriate descriptive statistics (ie, means and SDs, medians and IQRs or proportions) and 95% CIs constructed for the difference in outcomes between the control and intervention groups. To generate evidence of proof of principle, we will generate a range of CIs around the main estimate for the treatment effect to inform us of the likelihood of where the “true” estimate may lie and inform the power calculations for a definitive phase III trial. The analysis will be adjusted for key predictors, including job type, baseline hours of work, and stroke severity.

### Study Organization

Monash University supports trial organization, data management, and monitoring.

## Results

Funded in March 2019, organizational ethics authorization was provided in October 2019. Although the first participant was randomized in November 2019, COVID-19 restrictions have affected Work trial recruitment to date. Data collection was not complete at the time of manuscript submission. Expected results are to be published in January 2023.

## Discussion

### Anticipated Findings

Returning to work is important to stroke survivors [5]; however, more than 60% of working-age people in Australia who experience a stroke report an unmet need with regard to returning to work [4]. In fact, almost 40% of people report ongoing unemployment or underemployment following a stroke [1]. Prior research has shown that vocational rehabilitation enables return to work [2], but there has been little research to date into interventions to support people with returning to work after stroke. The Work trial will determine whether providing a tailored vocational rehabilitation intervention is feasible early after stroke, as well as estimate the potential benefit. Given this trial will be conducted during the COVID-19 pandemic, we acknowledge that flexibility in the delivery of the Work trial intervention using telehealth may be required. Recruitment may be affected by COVID-19–related government restrictions; changes to this protocol will be disclosed using the CONSERVE statement for reporting extenuating circumstances.

This paper describes the protocol of a pilot randomized controlled trial designed to estimate the feasibility and benefit of providing early vocational rehabilitation to stroke survivors. The data obtained in this trial will inform the development of a future clinical trial powered to detect clinically significant changes in the chosen outcome measures if feasible.

### Conclusions

This pilot feasibility study will provide necessary information regarding the delivery of vocational rehabilitation and the potential vocational outcomes experienced by people who have had a stroke. Findings from this pilot trial will provide data on the initial efficacy of an early vocational rehabilitation intervention and valuable feedback on the design and implementation of the intervention in the real world.

## Abbreviations

CONSERVE: CONSORT and SPIRIT Extension for RCTs Revised in Extenuating Circumstances
CONSORT: Consolidated Standards of Reporting Trials

## Data Availability

The data sets generated during this study are not publicly available due to ethics restrictions but are available from the corresponding author upon reasonable request and with permission provided by the Alfred Health Human Research Committee.
